# A Scoping Review of Health Education Programs for Improving Men’s Engagement with Health Services

**DOI:** 10.34172/hpp.45321

**Published:** 2026-08-21

**Authors:** Jalil Hosseini, Keshvar Samadaee Gelehkolaee, Sakineh Hajebrahimi, Samira Shariatpanahi, Fateme Tahmasbi, Neda Kabiri, Jiri Kantor, Hadi Mostafaei, Abtin Heidarzadeh, Hanieh Salehi-Pourmehr

**Affiliations:** ^1^Men’s Health and Reproductive Health Research Center, Shahid Beheshti University of Medical Sciences, Tehran, Iran; ^2^Sexual and Reproductive Health Research Center, Department of Reproductive Health and Midwifery, Faculty of Nursing and Midwifery, Mazandaran University of Medical Sciences, Sari, Iran; ^3^Research Center for Evidence-Based Medicine, Faculty of Medicine, Tabriz University of Medical Sciences, Tabriz, Iran; ^4^Center of Evidence-based Education and Arts Therapies: A JBI Affiliated Group, Faculty of Education, Palacký University Olomouc, Olomouc, Czech Republic; ^5^Department of Urology, Comprehensive Cancer Center, Medical University of Vienna, Vienna, Austria; ^6^Medical Education Research Center, Department of Community and Family Medicine. School of Medicine. Guilan University of Medical Sciences, Rasht, Iran

**Keywords:** Guideline, Health education, Health promotion, Health services, Men’s health, Scoping review

## Abstract

**Introduction:**

To map and synthesize evidence from health education programs (HEPs) aimed at improving men’s engagement with health services, including original studies, community programs, and clinical guidelines.

**Methods:**

Following PRISMA-ScR, we conducted a comprehensive search of databases and guideline repositories to identify (1) health education programs, (2) original studies evaluating HEPs, and (3) clinical guidelines related to men’s health. Data were synthesized thematically across these evidence streams.

**Results:**

Ninety-two sources were included (30 programs, 39 primary studies, 23 guidelines). Effective HEPs commonly used gender-sensitive messaging, decision aids, peer- or community-based delivery, and digital platforms. Programs situated in male-centric environments (sports clubs, barbershops, workplaces) demonstrated high acceptability and engagement. Guidelines consistently emphasized routine screening, particularly for prostate cancer, and lifestyle interventions such as physical activity and smoking cessation.

**Conclusion:**

Across diverse evidence types, HEPs that are culturally tailored, gender-sensitive, and delivered in male-friendly settings are most effective in increasing men’s engagement with health services. Strong emphasis on prostate cancer screening in existing evidence highlights the need for broader attention to other prevalent male health issues.

## Introduction

 Men’s health has emerged as a critical public health issue, characterized by significant disparities in health outcomes compared to women. Despite improvements in overall health, men continue to experience shorter life expectancies and higher rates of chronic diseases, largely due to socio-cultural factors and gender norms that discourage help-seeking behaviors. Addressing men’s health from a gender perspective is essential, as it reveals how societal expectations and inequalities contribute to these disparities. One critical aspect contributing to these disparities is the tendency for men to engage less frequently with health services.^1^ Men’s health issues are often overlooked in public health discussions, despite evidence showing they face higher mortality rates from non-communicable diseases and mental health crises.

 Studies indicate that men are less likely to seek preventive care, adhere to treatment regimens, and participate in health education programs. This reluctance can be linked to traditional gender norms that valorize stoicism and self-reliance, often discouraging men from expressing vulnerability or seeking help. The reluctance of men to seek medical help exacerbates health problems, leading to delayed diagnoses and poorer outcomes. Examining men’s health through a gender lens highlights the impact of masculinity on health behaviors, such as the stigma surrounding mental health and the cultural pressures to avoid seeking help. Gender inequalities in healthcare access and treatment further perpetuate these disparities, necessitating targeted interventions to improve men’s health outcomes. Even though a large corpus of health research suggests that men with equivalent socioeconomic disadvantages as women face poorer health outcomes in terms of disability, chronic diseases, injury rates, and death, men’s health is rarely investigated from the viewpoint of gender. Men are experiencing a pervasive, silent health crisis as a result of gender inequalities in health behaviors, preventative screening, and medical care.^2^ Poor health outcomes have also been noted in several studies, including delayed HIV antiretroviral treatment initiation and worse life expectancies for men than for women.^3-5^ The masculinity of men and other sociocultural factors have been connected to their distaste for medical services.^5,6^ Gender roles significantly influence not only how men perceive their health but also their willingness to engage with healthcare systems. By recognizing these dynamics, we can identify targeted strategies to improve men’s health engagement.

 Men’s health and well-being have recently drawn increasing attention in the European Region of the World Health Organization (WHO) due to the high prevalence of male premature death, especially in the eastern part of the Region. Therefore, a firm commitment and decisive action are necessary in light of the growing interest within civil society in issues pertaining to men’s health and the importance of males in the agenda for gender equality. The Sustainable Development Goals (SDGs) of the United Nations 2030 Agenda, including SDG 3 on good health and well-being, SDG 5 on gender equality, and SDG 10 on reduced inequalities, offer a strong framework for promoting action aimed at lowering premature mortality among men and improving men’s mental health and well-being.^7^ Health education programs (HEPs) have a history of raising people’s knowledge of their health and enhancing health outcomes. Scholars have documented how HEPs have encouraged people to adopt healthier lives in recent years.^8-16^ While the focus on women’s health is crucial, it is equally important to address men’s health needs through a comprehensive, gender-sensitive approach that acknowledges the unique challenges men face in accessing healthcare. This scoping review aims to systematically identify, characterize, and map the landscape of published HEPs designed to improve men’s engagement with health services. Specifically, it seeks to:

 Catalogue the range of existing HEP interventions targeting men’s health engagement.

 Characterize these interventions by their design (e.g., theoretical basis, delivery format, setting, duration), target population, and core educational components.

 Identify key gaps in the existing evidence base to guide future primary research and systematic reviews on intervention effectiveness.

## Methods

###  Review Approach and Rationale

 This scoping review was conducted based on the JBI framework for scoping reviews^17^ in order to map the breadth and characteristics of HEPs aimed at promoting men’s engagement with health services. The scoping review design was chosen due to the nature of the question and because of the wide range of available evidence, including randomized trials, observational studies, national programs, and clinical practice guidelines.

 This approach aligns with the PRISMA-ScR (Preferred Reporting Items for Systematic reviews and Meta-Analyses extension for Scoping Reviews).

###  Search Strategy

 The search approach used was to locate published or unpublished research. Three steps were used in the present review’s search process. An initial PubMed search was conducted, and then the title and abstract were examined based on the appropriate keywords according to the PCC. The search was conducted, using all the identified keywords and index terms, including but not limited to “Health,” “Education,” “Program,” and “Men’s health”, “sexual and reproductivity”, “prostate cancer”, and “HIV” and their synonyms, and also controlled vocabularies. The search strategy for retrieving guidelines and programs in Medline is available in the supplement file. The updated search was conducted for guidelines retrieved on the 15th of May 2023. The search strategy for the databases search are illustrated in Supplementary File 1.

 The current study has two stages of search to retrieve relevant evidence.

####  Stage 1: Search for Primary Studies and Health Education Programs

 For this purpose, in the first phase, a thorough literature search was carried out in Medline (via Ovid), Embase, PubMed, Scopus, and Web of Science databases. Google Scholar was also searched for any potentially relevant studies.

####  Stage 2: Search for Clinical Practice Guidelines

 In the next phase, any clinical practice guidelines, programs, recommendations, and checklists related to men’s health in the experiences of countries were searched. To search for clinical guidelines, recommendations, and a checklist related to men’s health, specialized sites related to clinical guidelines and relevant scientific associations were searched in PubMed, the TRIP database, National Institute for Health and Care Excellence (NICE), SIGN, Guidelines International Network (GIN), European Association of Urology (EAU), Guideline Central and WHO repository.

 Additional searches were performed in PubMed and Google to identify national or organizational guidelines, checklists, and health promotion programs.

 Gray literature sources (ProQuest, ResearchGate, university repositories, WHO Library, Grey.net) were searched for unpublished studies, theses, and reports.

###  Inclusion criteria

####  Population

 Adult men aged 20–65 years. This range was selected to focus on working-age men who face distinct barriers and patterns of healthcare under-utilization. Although health education for older men is important, this review targeted a specific demographic with unique engagement challenges.

####  Concept

 Health education programs (HEPs) or initiatives designed to increase men’s engagement with healthcare, including any components of the primary healthcare package:

- Health promotion (education, counselling)
- Disease prevention (screening, vaccination)
- Treatment-related education
- We considered healthcare utilization (e.g., screening uptake, visits), adherence to preventive behaviors, and health-related quality of life in the included evidence.

####  Context

 This scoping review considered all evidence across the globe in primary healthcare level.

####  Evidence Types

- Randomized controlled trials
- Quasi-experimental studies
- Observational studies
- Qualitative studies
- Mixed-method studies
- Clinical practice guidelines
- National programs

Exclusion Criteria:

- Studies exclusively involving men > 65 years^18^
- Articles lacking a health education component

###  Study selection

 All found citations were put into EndNote X9 after the search was finished, and duplicates were eliminated. Titles and abstracts were examined by two independent reviewers for evaluation in relation to the review’s inclusion criteria. Two independent professional reviewers thoroughly evaluated the whole text of the selected eligible studies in accordance with the inclusion criteria. If any full-text studies didn’t fit the inclusion criteria, they were excluded. Discussions between reviewers were used to settle any disputes.

###  Data extraction 

 Data from the studies that were included in the review were extracted by two independent reviewers using a modified version of the usual JBI data extraction tool. Authors, publication year, nation, research methodology, population (sample size, mean age), educational program, and results were gathered specifically from the qualifying studies. Any discrepancies were resolved through conversation or by the third reviewer.

###  Assessment of the methodological quality 

 Although scoping reviews do not require mandatory critical appraisal, we assessed methodological quality to support the interpretation of findings.

 In the review, eligible studies were critically evaluated by two independent reviewers at the study level for methodological quality using the standardized Joanna Briggs Institute (JBI) critical appraisal instruments, including the checklist for randomized controlled trials, quasi-experimental studies, cohort studies, and case series to determine the extent to which a study has addressed the possibility of bias in its design, conduct, and analysis. Any differences of opinion among the reviewers were settled through conversation or consultation with a third reviewer. Studies were not disqualified for having poor methodology or a high risk of bias.^19^

 To assess the quality of the guidelines included, we employed the Appraisal of Guidelines for Research & Evaluation (AGREE II) instrument. AGREE II is a widely used tool that evaluates guidelines across six domains: scope, stakeholder involvement, rigor of development, clarity, applicability, and editorial independence. Each domain contains several appraisal criteria (items), totaling 23 in all. Additionally, AGREE II includes two overall assessments: overall guideline quality and recommendation for use.^20^ A criterion of 60% was used to assess the overall quality of each domain’s final score on the AGREE II. Five domains with ratings of better than 60% were considered to be of outstanding quality, indicating that the guideline. When three or four of the domains scored at or above 60%, and when no more than two domains scored at or above 60%, the recommendations were categorized as being of medium quality and low quality, respectively.^21^ Each guideline’s overall score, as well as the domain scores, was determined.

 No studies or guidelines were excluded based on quality; however, appraisal results were used to contextualize the strength of evidence during synthesis.

###  Data Synthesis 

 Because the included evidence comprised diverse sources, health education programs, original empirical studies, and clinical practice guidelines, we used a descriptive thematic synthesis approach to map and organize key concepts across evidence streams.^22^ The synthesis proceeded in three stages:

 1. Within-stream coding

 Each evidence stream (programs, original studies, guidelines) was reviewed independently. Two reviewers extracted key elements:

- Target health issue
- Educational strategy or delivery method
- Target population
- Behavior change mechanisms
- Reported outcomes

Codes were inductively generated and grouped into preliminary themes within each evidence stream.

 2. Cross-stream comparison

 Codes from the three streams were compared to identify convergent themes. This step allowed us to assess:

- Whether themes from programs were supported by findings from RCTs or observational studies
- Whether guideline recommendations aligned with or differed from the interventions evaluated in studies
- Where gaps existed (e.g., guideline emphasis without supporting intervention evidence)

3. Integrative synthesis and thematic refinement

 Final themes were developed by triangulating evidence from programs, empirical studies, and guidelines.

 When discrepancies occurred (e.g., guidelines recommending screening but few programs delivering screening-focused education), they were explicitly noted in the results.

 This analytic process ensured a structured, theme-based synthesis that systematically mapped evidence across programs, empirical studies, and clinical practice guidelines.

## Results

###  Study inclusion 

 Totally, 10901 citations (9463 through the first phase of relevant databases, and 1190 in the phase of guidelines search) were identified by the electronic search, hand search, and reference check. After omitting duplicated citations, 5229 studies remained for the screening process. Out of these, 5117 studies were removed, and 112 studies and guidelines were selected based on titles and abstracts. In the full-text selection step, 20 studies were excluded; 12 studies were excluded for having insufficient data or an inappropriate population. The scoping review finally included 92 evidence types:

- 30 health education programs
- 39 original clinical studies
- 23 clinical practice guidelines

The PRISMA flow diagram (Figure 1) presents further details on the selection procedure.

**Figure 1:**
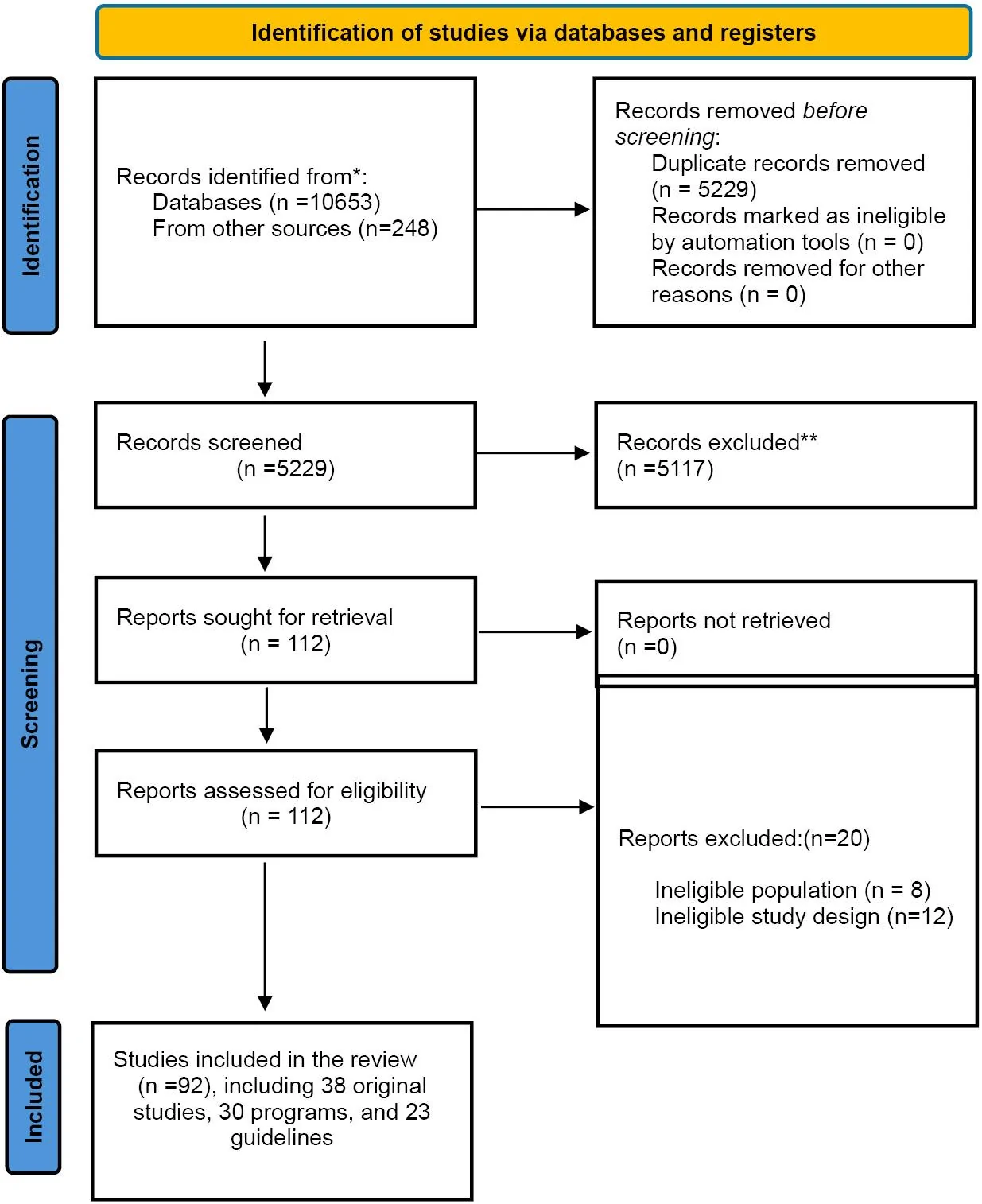
PRISMA flow diagram

###  Characteristics of included studies 

 The evidence base comprised diverse educational strategies, populations, and health conditions. We categorized the retrieved evidence into three tables (Tables 1, 2, and Supplementary File 2). Ninety-two evidence types, including 30 programs such as brochures, e-programs, and community intervention programs targeted at men’s health (Table 1), 39 RCTs, quasi-RCTs, or observational studies (Supplementary File 2), and 23 guidelines and checklists, which mainly or partially discuss men’s health (Table 2), were included in this study.

**Table 1:** Evidence summary included in the systematic review

**Study**	**Setting **	**Program**	**Description of program**
^23^	Miami, Denver and Baltimore, US	Community Voices’ three programs—Miami’s Overtown Men’s Health Study, Denver Men’s Health Initiative, and Baltimore Men’s Health Center	Assessment of men’s health care needs. Denver and Baltimore also designed and delivered services aimed at reducing barriers to health care access, providing primary care, and improving health outcomes for vulnerable men
^24^	UK	Man MOT, a suite of online health information and advice services aimed at men	Promotion of do-it-yourself health checks & challenges to get healthier. The 20-page full color A5 booklet, written by Jim Pollard with cartoons by John Byrne, full of simple, practical tips that improve the health of pretty much anyone. An anonymous and confidential live online text chat to an NHS GP service. There were also occasional additional issue-specific chat sessions, as well as an open-all-hours email enquiry service. Man MOT offered web-based male-targeted health information in a more traditional magazine-style format and a ‘Find local services’ search facility
^25^	Australia	GPs4Men, the Australian GPs Network for Men’s Health	The Australian GPs Network for Men’s Health (GPs4Men) was formed in response to a lack of policy and funding for men’s health, and the absence of a mechanism for GPs and divisions of general practice to exchange information and ideas about men’s health. The mission of GPs4Men is to improve the health of men both nationally and individually.
^7^	WHO, Europe	Strategy on the health and well-being of men in the WHO European Region	The goal of the strategy is to improve men’s health and well-being through evidence-informed, gender-responsive and equity-driven approaches that transform the gender roles, norms and structures that affect men’s exposure to risk factors and act as a barrier to gender equality and health equity achievements in Europe.
^26^	Uganda	Sexual Reproductive Health and Rights Project (SRHR)	Project contributing to improved sexual and reproductive health in Uganda through increasing access and use of quality, inclusive and integrated SRHR and HIV services and by promoting positive healthy sexual relationships that will contribute to reducing new HIV infections.
^27^	Kenya	Kenya's voluntary medical male circumcision program	The goal of the Kenya National Strategy for Voluntary Medical Male Circumcision is to contribute to the reduction of new HIV infections among men by providing integrated, safe and accessible voluntary male circumcision services.
^28^	Midwest University, Wentzville, Missouri	Man Up Monday	This campaign sought to increase awareness of sexual health and chlamydia testing; motivate students, particularly sexually active men who do not pursue regular sexually transmitted infection (STI) testing, to get tested; and improve the capacity of the student health center to provide free chlamydia testing and treatment for all students
^29^	Australia	*Man Up *multimedia intervention	The aim of the concept test was to increase understanding of effective visual and written communication with Australian men in the context of suicide and masculinity and test and explored the general functionality and appeal of the website to Australian men.
^30^	Canada	Check-Mate tool	Check-Mate tool, as part of a global evaluation of the Movember Foundation’s Social Innovators Challenge, provides an initial set of evidence-based guidelines for incorporating gender-related influences in men’s mental health promotion programs.
^31^	Canada	HOMEBASE	For Men living with chronic pain, participants are offered a variety of programming choices including access to peer mentors, opportunities to connect socially through activities such as fitness and music, as well as opportunities to engage with and serve their communities
^32^	Canada	Dad HERO	For Incarcerated and ex-offender fathers, an 8-week in-prison parenting course as well as ongoing in-prison and community-based support groups for incarcerated and ex-offender fathers.
^33^	Australia	Entourage	For Young men with social anxiety, development and distribution of an interactive e-mentoring program using the moderated online social therapy platform
^34^	United Kingdom	The Changing Room	For Men in their middle years, participants are engaged men in discussions about mental health through their interest in soccer, physical activities, talking groups, and other opportunities to socialize
^35^	United Kingdom	Men’s Pie Club	For Socially isolated men from a variety of communities, men come together to bake pies and have the opportunity to socialize
	Canada	Apocalypse Made Easy	For Male IT workers, participants sign up to take part in interactive walking tours that require working together and role-playing through post-apocalyptic-themed scenarios and solving challenges
^36^	United Kingdom	Brothers Through Boxing	For young men who are not in employment, education, or training, participants engage in a boxing-themed fitness program and activities designed to be self-reflective around themes of social connectedness and well-being
^37^	United Kingdom	Ex-Cell 50+	For older ex-offenders, D/deaf ex-offenders, homeless men, using a “Self-Reliant Group” group development methodology, participants are encouraged to collectively save funds and start a microbusiness. They are offered opportunities to receive business mentorship and learn consensus and decision-making skills.
^38^	Australia	Vet Connect	For male army veterans, participants take part in three weekend retreats that sometimes involve their families as well as teleconference “catch-ups” and have access to a facilitated Facebook group.
^39^	Australia	Dad’s Group Inc	For new fathers, meet at a location close to their homes and have the opportunity to interact through facilitated social media groups.
^40^	Australia	WOW Sand and Surf	For young men, a 6-week learn-to-surf wellness program. Group meetings include facilitated discussions around wellness and connection as well as mentored surfing time with mental health–trained surf instructors.
^41^	Uganda	Networks project	The aim of the project was to mobilize and strengthen groups of people living with HIV, and enable them to access HIV services at a time when HIV stigma was rampant
^42^	Canada	“Don’t Change Much” e-Program	“Don’t Change Much” e-Program launched by the Canadian Men’s Health Foundation, inspire and equip men and their families to lead healthier lives. Text, video, and audio information is purposefully delivered to improve diet, exercise, sleep, and stress manage- ment as well as reduce alcohol use and/or smoking.
^43^	Australia	The Aboriginal Men's Health Implementation Plan	The aims of this projects are: increase the number of Aboriginal men employed within the health system and Aboriginal medical services; increase in the number of Aboriginal men using primary health services; development of an infrastructure aimed at improving access and acceptability of health services for Aboriginal men; increase the number of Aboriginal men’s support groups; increase the provision of outreach services for Aboriginal men; development of a memorandum of understanding between area health services and local Aboriginal medical services concerning the sharing of resources and collaboration in promoting health services to Aboriginal men; increase the use, by Aboriginal men, of the Isolated Patients’ Travel and Accommodation Assistance Scheme (IPTAAS).
^44^ ^45^	Global	HOPE Social Media Intervention for Global HIV Prevention	The HOPE (Harnessing Online Peer Education) study tests the feasibility, acceptability, and effectiveness of using social networking sites (specifically Facebook) to increase HIV prevention and testing.
^46^	UK	Implementation intentions	To promote performance of testicular self-examination in young males.
^47^	Southeast Michigan, US	Get Connected! Program	Seeking to promote HIV/STI testing ("Get Connected!") among young men who have sex with men.
^48^	El Paso, US	A community-based intervention for Hispanic men to encourage informed decision making about prostate cancer screening	Materials included Men’s Guides, short booklets that presented information in the same sequence as the slides and video clips. The learning objectives were: recognize that a health- related decision about screening needs to be made; choose their preferred role in this decision; engage in a decision-making process for prostate cancer screening; seek consultation with important others; discuss their screening decision with their health care provider; base their decision on information including characteristics of the test and personal preferences; and act on their decision.
^49^	South Wales, UK	Prosdex, Web-based PSA decision aid	Prosdex was developed as part of the UK Prostate Cancer Risk Management Program in order to help men make such informed decisions. Men considering the prostate specific antigen (PSA) test for prostate cancer, an increasingly common male cancer, are encouraged to make informed decisions, as the test is limited in its accuracy and the natural history of the condition is poorly understood.

**Table 2:** Guidelines and recommendations for men’s health

**Guideline/ recommendations**	**Health Screening**	**smoking and alcohol use**	**Nutrition**	**Physical activity**	**Sleep Apnea**	**Mental Health (depression)**	**Dementia**	**Eye examination**	**Testosterone Deficiency**	**Osteoporosis**	**LUTS**	**Work safety**	**Cardiovascular issues**	**HTN**	**Vaccination**	**abdominal aortic aneurysm**	**Diabetes**	**COPD**	**STDs**	**Cancers**	**Comments **
^50^	*		*	*	*	*			*	*	*									*	-
^51^																				*	prostate cancer
^52^	*		*	*		*						*	*							*	Especially prostate and bowel cancer
^53^	*	*	*	*													*			*	Prostate cancer
^54^																			*		HIV prevention
^55^						*	*	*		*			*			*	*	*	*	*	Aspirin use for cardiovascular diseases prevention, bladder, colorectal, lung, skin, and pancreatic cancer, HIV
^56^	*								*	*			*	*	*				*	*	PSA for prostate cancer
^57^	*	*				*													*		
^58^																				*	Prostate cancer screening and treatment
^59^	*	*	*	*		*	*				*		*	*			*		*	*	Urologic and colorectal cancers
^60^							*			*	*		*	*		*	*		*	*	Prostate and colorectal cancers
^61^									*		*									*	Prostate problems
^62^	*										*		*								
^63^																				*	PSA test (Prostate cancer)
^64^																				*	Prostate cancer
^65^																				*	Secondary Care Diagnosis and Management of Prostate Cancer
^66^																					Erectile Dysfunction
^67^											*										
^68^																				*	Prostate cancer
^69^	*	*		*									*							*	
^70^																				*	Pelvic floor muscle exercises for prostatic cancer

 Reducing premature mortality among men owing to non-communicable illnesses and accidental and intentional injuries, as well as promoting health and well-being among men of all ages, are the main objectives of the WHO strategy, which are intrinsically related to the aims of the SDGs and Health 2020; in addition, systems and policies that encourage men’s involvement in self-care, parenthood, accidental injury avoidance, and other activities reduce disparities across and within the nations of the area while also enhancing gender equality.^7^

####  A. Health education programs targeting men’s health 

 All the retrieved programs were published from 2001 to 2023, and many of them were conducted in Australia (8/30) and the United Kingdom (7/30). Based on the thematic synthesis of 30 health education programs, three main, distinct, interconnected themes were found, which are investigated in Figure 2. More details are described in Table 1.

**Figure 2:**
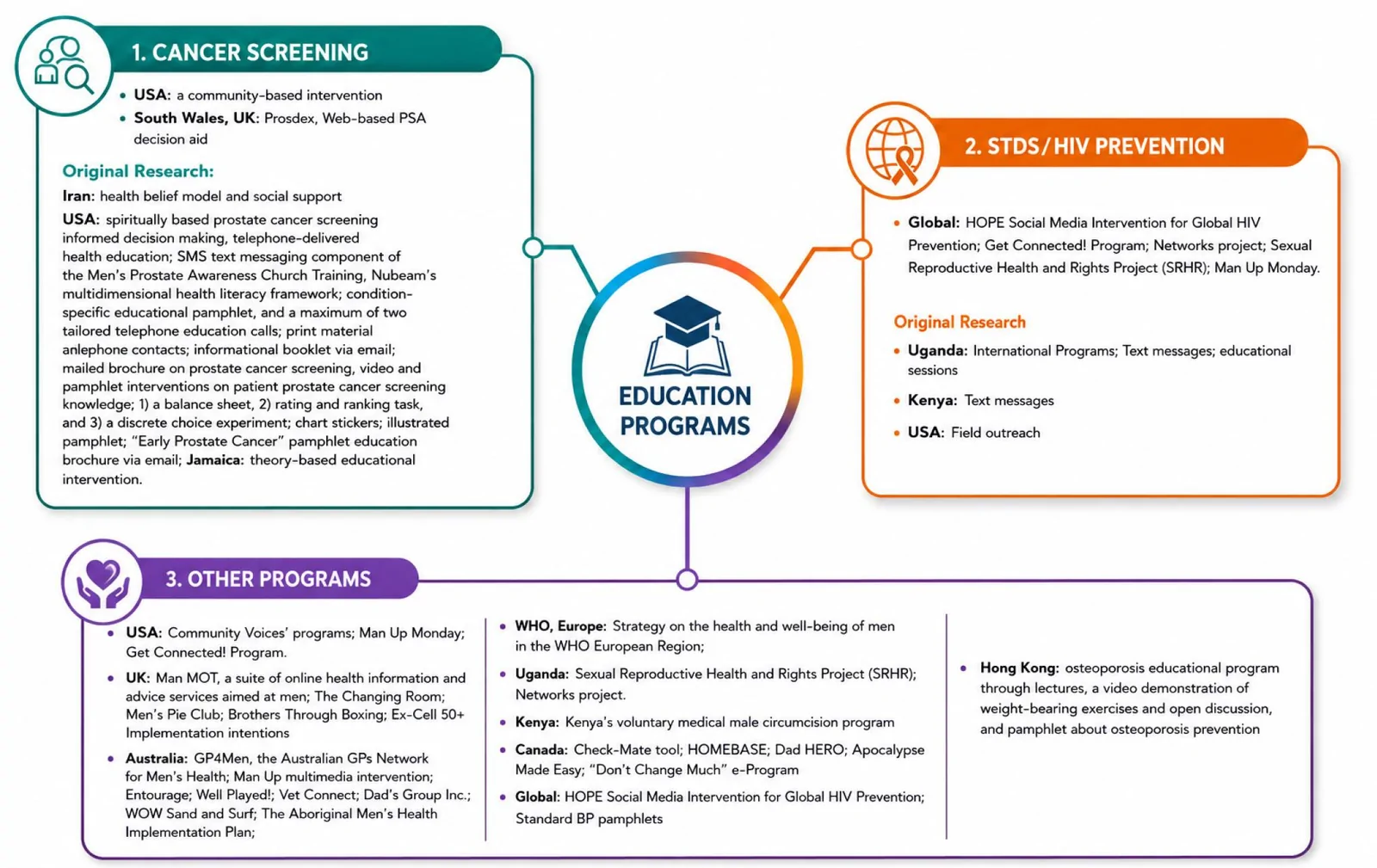
The programs targeting men’s health

####  Theme 1: Gender-Sensitive, Culturally Tailored, and Community-Based Programs

#####  Evidence from Programs and Studies

 Many programs designed for men particularly minority, Indigenous, and at-risk groups successfully incorporated culturally tailored messaging, male role models, and community settings (e.g., barbershops, churches, universities, prisons, and sport clubs). These programs increased engagement with PHC services, screening, HIV testing, and mental health resources.

 Examples include:

- Aboriginal men’s primary care initiatives
- The Man-Up multimedia mental health intervention
- The Prostate Cancer Awareness Church Training project
- Community-based HIV/STI testing campaigns

#####  Alignment with Guidelines

 Guidelines strongly emphasize culturally adapted communication and targeted outreach for high-risk male populations. Our synthesis shows consistent alignment between guideline recommendations and strategies in health education programs in this area.

####  Theme 2: Decision Aids and Informed Decision-Making Tools (especially for prostate cancer)

#####  Evidence from Programs and Studies

 A substantial portion of empirical studies and programs focused on decision-making about prostate cancer screening.

- RCTs^71-75^ showed that decision aids, pamphlets, videos, and balance sheets consistently improved men’s knowledge and decision quality.
- Community-based interventions using SMS or digital tools increased engagement and retention.
- Programs such as Prosdex provided web-based interactive education.

#####  Alignment with Guidelines

 Guidelines uniformly recommend patient-centered decision-making for prostate cancer screening and emphasize informed choice rather than routine PSA testing.

 Strong concordance exists between guideline emphasis and the substantial empirical evidence supporting decision aids.

####  Theme 3: Sexual Health and HIV/STI Prevention Programs

#####  Evidence from Programs and Studies

 Seven programs and multiple original studies addressed sexual health, HIV prevention, and STI testing.

 Key strategies included:

- Motivational interviewing (effective in RCTs with African-American MSM)
- Community sensitization for partner testing
- Digital outreach and social media interventions
- Comprehensive S/RHR programs (e.g., Uganda SRHR project)

Outcomes included increased HIV testing uptake, STI screening, improved conversations about family planning, and enhanced sexual wellbeing in men with chronic conditions (e.g., SCI).

#####  Alignment with Guidelines

 Guidelines consistently emphasize HIV/STI screening among high-risk men, partner testing, and sexual health counseling.

 High consistency was observed between recommended practices and evaluated interventions.

####  Theme 4: Primary Healthcare Engagement and Chronic Disease Prevention

#####  Evidence from Programs and Studies

 Several studies focused on barriers to PHC access, utilization of services, cardiovascular health education, diabetes management, and chronic disease self-care.

 Programs for vulnerable men (veterans, homeless men, unemployed men) improved attendance and engagement.

#####  Alignment with Guidelines

 Guidelines extensively recommend:

- CVD risk screening
- Diabetes screening
- Lifestyle modification (physical activity, smoking cessation, alcohol reduction)

However, empirical evidence for large-scale educational interventions in these areas was less frequent, highlighting a gap between guidelines and available programs.

####  Theme 5: Mental Health Literacy and Help-Seeking Promotion

#####  Evidence from Programs and Studies

 Five programs focused on encouraging mental-health help-seeking and reducing stigma (e.g., Man Up, Check-Mate, The Changing Room).

 Outcomes included improved mental health literacy, reduced stigma, and increased service engagement.

#####  Alignment with Guidelines

 Guidelines frequently recommend depression screening, suicide risk assessment, and early identification of mental health disorders in men.

 This alignment reinforces the importance of expanding mental health–focused HEPs.

####  Guideline Synthesis

 Twenty-three guidelines collectively highlighted:

- Prostate cancer and colorectal cancer screening
- HIV/STI testing
- CVD and metabolic disease prevention
- Mental health assessment
- Lifestyle modification

These guideline recommendations largely aligned with evidence from studies and programs in cancer, HIV, and mental health, but less so for chronic diseases and aging-related conditions, revealing key gaps.

*A.1. Promoting primary health care (PHC): *The objective of these papers, which were directed at various vessels, was the promotion of PHC. For instance, one study aimed to raise the percentage of Aboriginal males using PHC services, the percentage of Aboriginal men working in the health system, and the percentage of Aboriginal men receiving medical care.^43^ In another study, Denver and Baltimore developed and provided services targeted at lowering obstacles to accessing healthcare, offering primary care, and enhancing health outcomes for vulnerable men.^23^ The establishment of the GPs Network for Men’s Health (GPs4Men) in Australia, in response to the lack of financing and policy for men’s health, as well as the lack of a system for GPs and general practice divisions to share knowledge and opinions regarding men’s health, was described by another study.^25^ Enhancing the process of decision-making was the purpose of three other programs. In Ex-Cell 50+ program, taking place in the UK, older ex-offenders were provided to obtain business with mentoring and gain consensus-building and decision-making abilities.^37^ Prostate cancer screening decision-making was encouraged through a community-based initiative for Hispanic men.^48^ A web-based decision aid and a part of the UK Prostate Cancer Risk Management Program called Prosdex was developed to assist men in making such educated decisions.^49^

*A.2. Targeting different conditions: *Several studies targeted diverse conditions and/or populations. For instance, men of minority or at-risk groups were the subject of 7 studies, including young homosexual men^47^, unemployed or uneducated young men^36^, homeless men^37^, ex-offenders^32,37^, army veterans^38^, and Aboriginal men (which was reported in the previous paragraph).^43^

 Seven studies investigated various aspects of men’s sexual health. Sexual Reproductive Health and Rights Project (SRHR) in Uganda aimed to reduce new HIV infections by expanding access to and use of high-quality, inclusive, and integrated SRHR and HIV services, as well as by fostering good, healthy relationships.^26^ The Kenya National Strategy for Voluntary Medical Male Circumcision aims to provide comprehensive, safe, and accessible voluntary male circumcision services in order to reduce new HIV infections among males.^76^ Man Up Monday campaign was held to raise awareness of sexual health issues and chlamydia testing and to encourage students to get tested, especially sexually active men who don’t regularly get tested for STIs.^28^ In another project in Uganda, the goal was to empower and bolster communities of individuals living with HIV so they could access services during a period when HIV stigma was pervasive.^41^ Two studies focused on the HOPE Social Media Intervention for Global HIV Prevention.^44^ Lastly, the “Get Connected!” program promoted HIV/STI testing in men who experience same-sex relations.^47^

 Another common subject among the included research was the promotion of mental health, which was addressed in 5 studies by various initiatives, including the Man Up multimedia intervention,^29^ Check-Mate tool,^30^ Entourage, ^33^ The Changing Room,^34^ and “Don’t Change Much” e-Program.^42^

 Raising cancer awareness was conducted through different methods; including encouraging adolescent guys to self-examine their testicles,^46^ guiding Hispanic men to make educated decisions about prostate cancer screening^48^, and using the PSA decision support tool Prosdex, which is accessible online.^49^

 Finally, men suffering from chronic illnesses were provided with several programs; for example, having access to peer mentors and the opportunity to socialize through activities like exercise and music.^31^

#####  B. Original studies

 Studies in this category were published from 1999^77^ to 2021.^78^ Studies were conducted mostly (17/38) in the United States, with various African regions coming in second. (8/38) and then in Australia (6/38) after that. This category used a variety of study designs, including clinical trials and qualitative analysis. Supplementary File 2 provides a more thorough overview of the research design.

 Some of the studies that were reviewed focused on a particular group of men, including African-Americans^71,73,79-81^ or black men^82,83^, Aboriginal and Torres Strait Islanders^84^, veterans^75^, soldiers^85^, metal workers^86^, men suffering from spinal cord injury^87^, homeless men^88^, and men who have sex with men (MSM).^81^

 The promotion of cancer awareness and sexual health emerged as the most frequent themes from the studies that were considered; these topics will be covered in more detail in the paragraphs that follow. Other programs focused on the following subjects: cardiovascular health^83,86^, utilization and challenges of PHC^84,88-94^, psychological or mental health^95,96^, and encouraging a healthy lifestyle.^97,98^

*B.1. Prostate cancer:* Increasing public knowledge of cancer, particularly prostate cancer (14/38) was the primary focus of several studies, which were carried out using a variety of methods, including community education. After the educational intervention that will be looked at in the following paragraphs, the majority of this research reported promising findings.^71-75,77-79,82,94,99-101^

 In order to determine how a prostate cancer screening decision-aid might improve patients’ comprehension, attitudes toward, and usage of prostate cancer screening tests, Schapira and VanRuiswyk performed an RCT in the year 2000. Their research showed how successfully an illustrated leaflet increased awareness of prostate cancer, while having no negative effects on the use of screening examinations.^74^

 In 2004, Partin et al. carried out a further RCT to evaluate the impact of video and pamphlet treatments in 893 male veterans. Patient education and self-reported decision-making were improved by mailed treatments, which also changed screening preferences. The efficacy of the pamphlets and video treatments examined was equivalent.^75^

 The role of these educational interventions in enhancing the informed decision-making process was also investigated in Myers et al.’s work from 2005^71^ to 2011.^72^ More creatively, the Men’s Prostate Awareness Church Training project, a men’s health intervention for African American males, was examined by Le et al. to see whether it was feasible and acceptable to add short message service (SMS) to the initiative. These texts’ main goals were to keep workshop attendees’ memories fresh, to provide follow-up support, to spread inspirational and spiritual ideas, and to keep them engaged. This study’s findings suggested that improving health activities targeted at adult African American men might be accomplished in part through receiving SMS.^73^ Men were randomly assigned to one of three groups in the Pignone et al. RCT, depending on the type of clarification method they would receive: a balance sheet (n = 302), a rating and ranking task (n = 307), or a discrete choice experiment (n = 302). They then decided which traits were most crucial, ranking them from most important to least important.^101^ More than 40% of those surveyed admitted to recently having a PSA test, according to the findings. The balance sheet (35.1%) and the discrete choice experiment (32.5%) were given to participants, and those who completed the rating and ranking assignment were more likely to rank minimizing the chance of dying from prostate cancer as their most important goal (54.4%) (*P* < 0.001). The unlabeled PSA-like option (as opposed to the “no screening”-like option) was more preferred by those who received the balance sheet (43.7%) than by those who received the rating and ranking (34.2%), the discrete choice experiment (20.2%), or the rating and ranking plus balance sheet (33.4%). Three different studies’ findings revealed that a significant proportion of respondents wanted to undergo a PSA test (balance sheet, 77.1%; rating and ranking, 76.8%; and discrete choice experiment, 73.5%; P = 0.73).^100^ The experimental group in a more recent quasi-experimental study by Khani Jeihooni et al, which included 200 Iranian men (100 in the experimental group and 100 in the control group), got six training sessions as part of the educational intervention. In terms of knowledge, perceived susceptibility, perceived severity, perceived advantages, self-efficacy, signals to action, social support, and TSE performance, the experimental group considerably outperformed the control group six months following the intervention.^78^

 Further details are provided in Supplementary File 2.

*B.2. Sexual health:* Despite having a similar focus on men’s sexual health, the research in this area varied widely in its approaches.^26,81,87,102-104^

 The hypothesis that motivational interviewing, as opposed to traditional field outreach, increases HIV counseling and testing among young African-American MSM, as well as rates of return for test results, was put to the test in the RCT by Outlaw et al.^81^ The use of motivational interviewing to field outreach effectively encouraged this demographic, who are at high risk for HIV, to learn their status. Additionally, the authors stated that by using evidence-based short counseling techniques and focusing on high-risk minority communities, peer outreach workers can be successfully educated to minimize health inequalities.^81^

 In order to examine the function of community sensitization in the prevention of STDs, Mohala et al. carried out an RCT in which pregnant women were given a written invitation for their male sexual partner to take advantage of pair voluntary counseling and testing (VCT) for HIV and engage in prenatal care. According to their findings, more men are taking advantage of the couple VCT and receiving prenatal care.^102^

 The study by Kabagenyi et al. examined whether conversations about family planning with a health professional were a major determinant in sexually active men’s use of modern contraceptives and men’s reporting of partner contraceptive usage.^103^ Modern contraceptive usage was strongly correlated with men discussing family planning with healthcare professionals. They suggested that it may be necessary to create chances for men to continue participating in family planning issues at the levels of health facilities and communities, due to the significant role that conversations with health workers play in forecasting their participation in such concerns.^103^

 More broadly, the cross-sectional study by Chirau et al. examined risky sexual behavior in males who underwent adult circumcision. The risky activities were 20% more common than average. The incidence of many partnerships and payment for sex was greater than the national norm, indicating risk compensation.^104^

 The study by Stern et al. looked at a three-year intervention project that was carried out in the Hoima region of Uganda with the goal of including guys in sexual and reproductive health as clients, equal partners, and change agents. After the intervention, more men used sexual health services and helped their spouses use them. They also reported being more knowledgeable about sexual and reproductive health, saying they helped with household chores and contraceptive decisions, and showing less tolerance for domestic violence.^26^

 In an RCT, Zarei et al. assessed the effects of a mobile-based educational intervention on sexual marital life in Iranian men with spinal cord injury (SCI). After 4 weeks and 8 weeks from the baseline, there was a significant difference in the between-group mean of sexual adjustment and marital satisfaction (*P* < 0.001).^87^

 Finally, the Tung study showed that an osteoporosis education program may be successful in enhancing men’s and women’s knowledge and health attitudes regarding osteoporosis and related preventative practices. Although implementing these programs in nursing to encourage men to undertake osteoporosis preventive behaviors is generally advised, it did not increase men’s self-efficacy in completing such activities.^105^

 Further information is provided in Supplementary File 2.

#####  C. Guidelines or recommendations 

Table 2 is a summary of the guidelines regarding male health. In general, 9/23 guidelines recommended routine health screening methods in men, to timely diagnose and improve their health condition. Cancers, especially prostate cancer, were the most discussed health issues in the guidelines regarding men’s health. Guidelines recommended screening tests such as PSA and colonoscopy for early detection of malignancies in 16 out of 23 guidelines. In addition to malignancies, LUTS was the other health condition recommended to be followed strictly in men in the 6 guidelines. The second most reported health problem was STDs, including HIV, which was reported in 6 guidelines. The mental health of men and screening for mental disorders such as depression were another point of interest, which are recommended in 5 guidelines. In addition, the neurophysiological evaluation of men for assessment of dementia risk was also considered in 3 guidelines. Cardiovascular disease (CVD), diabetes, osteoporosis, testosterone deficiency, hypertension, and abdominal aortic aneurysm were also the other conditions that were considered in the guidelines. Regarding lifestyle, guidelines emphasize physical activity (5/23), smoking and alcohol abuse prevention methods (4/23), and nutritional interventions (4/23) as the main factors.

###  Methodological quality assessment

 The quality assessment results of eligible studies are shown in Table 3. According to the results, most of the included studies were of medium quality. Nevertheless, we excluded none of the studies due to low quality.

**Table 3:** Quality Assessment based on Joanna Briggs Institute Risk of Bias Tool (https://jbi.global/critical-appraisal-tools)

**Study ID**	**Q1**	**Q2**	**Q3**	**Q4**	**Q5**	**Q6**	**Q7**	**Q8**	**Q9**	**Q10**	**Q11**	**Q12**	**Q13**
Holt. 2010	UC	UC	NA	NA	NA	NA	Yes	Yes	Yes	Yes	Yes	Yes	UC
Myers, 2005	Yes	UC	Yes	No	No	No	Yes	Yes	Yes	Yes	Yes	Yes	UC
Outlaw, 2010	Yes	Yes	Yes	No	No	No	Yes	Yes	Yes	Yes	Yes	Yes	UC
Lepore, 2012	Yes	Yes	Yes	No	Yes	Yes	Yes	Yes	Yes	Yes	Yes	Yes	UC
Victor, 2011	Yes	Yes	Yes	No	No	Yes	Yes	Yes	Yes	Yes	Yes	Yes	UC
Partin, 2004	Yes	Yes	Yes	No	Yes	Yes	Yes	Yes	Yes	Yes	Yes	Yes	UC
Mohammadian, 2020	UC	UC	Yes	UC	UC	UC	Yes	Yes	Yes	Yes	Yes	Yes	UC
Ganassin, 2016	UC	UC	Yes	NA	NA	NA	Yes	Yes	Yes	Yes	Yes	Yes	UC
Zarei, 2020	Yes	Yes	Yes	NA	NA	NA	Yes	Yes	Yes	Yes	Yes	Yes	UC
Badger, 2013	UC	UC	Yes	NA	NA	NA	Yes	Yes	Yes	Yes	Yes	Yes	UC
Duncan, 2014	Yes	Yes	Yes	UC	No	No	Yes	Yes	Yes	Yes	Yes	Yes	UC
Wilt, 2001	Yes	Yes	Yes	NA	NA	NA	Yes	Yes	Yes	Yes	Yes	Yes	UC
Schapira, 2000	UC	UC	Yes	NA	NA	NA	Yes	Yes	Yes	Yes	Yes	Yes	UC
Myers, 2011	UC	UC	Yes	NA	NA	NA	Yes	Yes	Yes	Yes	Yes	Yes	UC
Mohlala, 2011	Yes	Yes	Yes	NA	NA	NA	Yes	Yes	Yes	Yes	Yes	Yes	UC
Young, 2013	Yes	Yes	Yes	Yes	Yes	Yes	Yes	Yes	Yes	Yes	Yes	Yes	UC
Ghanotakis, 2017	Yes	Yes	Yes	Yes	Yes	Yes	Yes	Yes	Yes	Yes	Yes	Yes	UC
Odeny, 2012	Yes	Yes	Yes	Yes	Yes	Yes	Yes	Yes	Yes	Yes	Yes	Yes	UC
Tung, 2006	Yes	UC	Yes	UC	UC	UC	Yes	Yes	Yes	Yes	Yes	Yes	UC

Q1. Was true randomization used for assignment of participants to treatment groups? Q2. Was allocation to treatment groups concealed? Q3. Were treatment groups similar at the baseline? Q4. Were participants blind to treatment assignment? Q5. Were those delivering the treatment blind to treatment assignment? Q6. Were treatment groups treated identically other than the intervention of interest? Q7. Were outcome assessors blind to treatment assignment? Q8. Were outcomes measured in the same way for treatment groups? Q9. Were outcomes measured in a reliable way? Q10. Was follow up complete and if not, were differences between groups in terms of their follow up adequately described and analysed? Q11. Were participants analysed in the groups to which they were randomized? Q12. Was appropriate statistical analysis used? Q13. Was the trial design appropriate and any deviations from the standard RCT design (individual randomization, parallel groups) accounted for in the conduct and analysis of the trial?

 For each guideline, the final overall quality as well as the sum of the scores for each AGREE II domain were calculated. In this review, clinical recommendations for men’s health were included. Using the AGREE technique, the included guidelines were evaluated. The overall score of the guidelines was between 4 and 6 of 7. Based on the primary agreement among authors, guidelines were concluded to have moderate methodological quality.

## Discussion

 This scoping review mapped and synthesized findings from HEPs, primary studies, and clinical practice guidelines to understand how best to engage men with health services. The review revealed several consistent patterns across evidence types regarding effective settings, delivery methods, health focus areas, and implementation gaps.

###  Effective Settings and Delivery Strategies for Engaging Men

 Across programs and empirical studies, male-friendly settings, particularly sports clubs, barbershops, community centers, and workplaces, were associated with higher engagement and program completion. These environments align with men’s everyday routines and foster familiarity, social identity, and peer accountability. Sports clubs, in particular, showed strong potential for engaging high-risk men, with interventions leveraging peer relationships and team identity to support health behavior change. Similarly, peer-led, community-embedded, and culturally tailored approaches consistently demonstrated high acceptability, especially among African American men, Indigenous men, and men who have sex with men. Programs that incorporated trusted community members, faith-based leaders, or peer mentors showed higher uptake of screening, HIV testing, and mental health services compared to clinic-based health education alone.

 Digital tools, including web-based decision aids, mobile messaging, and social media interventions, were also effective, particularly when anonymity, autonomy, and flexible access were important for participants. Digital delivery may be especially valuable in areas where masculine norms discourage help-seeking.

###  Overemphasis on Prostate Cancer Screening and Implications for Men’s Engagement

 A striking finding was the disproportionate focus on prostate cancer across programs, empirical studies, and guidelines. This reflects long-standing clinical debate around PSA testing and the cultural prominence of prostate cancer awareness campaigns. While decision-aid studies show clear improvements in knowledge and informed decision-making, this dominance raises concerns about the narrowness of current HEP efforts.

 Men’s health needs are broad, spanning cardiovascular disease (CVD), mental health, metabolic disorders, musculoskeletal health, substance use, and sexual health, yet very few educational programs target these conditions. Guidelines, in contrast, emphasize a broad suite of screening and prevention priorities, including CVD, diabetes, depression, cognitive assessment, and lifestyle counseling.

 The mismatch suggests that educational programming is not fully aligned with the broader goal of “men’s engagement.” Expanding HEPs beyond prostate cancer could better reflect the spectrum of men’s health needs and achieve greater public health impact.

 Our systematic review mapped the evidence for HEPs in encouraging men’s engagement with healthcare services. Three key themes emerged:

*Routine Health Screenings:* Guidelines consistently recommend routine health screenings for men, with a particular emphasis on cancer and STDs. Prostate cancer was the most frequently discussed health issue, with screening tests like PSA and colonoscopy recommended. Mental health screening, including depression, was also highlighted, along with neurophysiological evaluations for dementia risk. Other conditions addressed included CVD, diabetes, osteoporosis, testosterone deficiency, hypertension, and abdominal aortic aneurism.

*Lifestyle Interventions:* Guidelines consistently emphasized lifestyle modifications, including physical activity, smoking cessation, alcohol reduction, and nutritional interventions.

*Programmatic Approaches:* Studies explored various approaches to engaging men in health programs, such as targeting specific subgroups (African Americans or black men, Aboriginal and Torres Strait Islanders, veterans, and soldiers, metal workers, men suffering from spinal cord injury, homeless men, and men who have sex with men (MSM)), focusing on particular health conditions (e.g., cardiovascular health, mental health), and utilizing diverse delivery methods (e.g., web-based tools, peer mentoring).

 While the quality of many guidelines was moderate, our review identified several innovative approaches to men’s health education. For this purpose, we provided a comprehensive search to find the original studies targeting men’s health programs. These findings contribute to existing knowledge by highlighting the importance of routine health screenings, lifestyle interventions, and targeted programmatic approaches.

 Research shows that in many countries, women are more likely than men to utilize health services, albeit this difference may be explained by the fact that women use more services during their reproductive years.^106^ For instance, women saw general practitioners much more frequently than males from 15 to 80 years old in England in 2008 and 2009; the highest gender disparity was found in the 20- to 44-year-old age range.^107^ In addition, a Lithuanian study of middle-aged university staff members discovered that women were substantially more likely than males to get regular dental checkups.^108^ Studies done in Malawi, South Africa, Uganda, and Zimbabwe reveal that views about masculinity not only increase the risk of obtaining the human immunodeficiency virus (HIV), but also discourage males from getting tested for the infection, accepting their HIV-positive status, listening to nurses, and taking part in behaviors that promote good health.^109^ According to Cornell et al. findings, there is a “blind spot” in our understanding of men and antiretroviral therapy in Africa. These researchers point out, for instance, that men access antiretroviral therapy in Africa at a far lower rate than women, men start antiretroviral therapy later than women in the course of the illness, and they are more likely to stop treatment and disappear from follow-up.^110^ According to White et al.^111^ there should be three goals for public and governmental initiatives to promote men’s health. The first is in schools, where gender preconceptions of men can be challenged. The second is promoting the well-being and pleasure of men at work. A third essential area for policy is focusing on the health needs of disenfranchised men, men from minority populations, and men who have sex with other men, all of whom have a higher burden of disease and early mortality than other men. Recent years have seen the emergence of three different types of interventions aimed at men: outreach, collaboration, and gender transition. All three of these strategies are now supported by evidence. Interventions often involve outreach programs focused on males at pubs and bars, sports clubs, barbershops, schools, and the workplace, with a focus on weight loss, quitting smoking, and other lifestyle changes and other public places in high-income countries (including Australia, the United States, and western European countries). A second strategy entails working together with males to enhance the health of women and children. According to Ghanaian study, child vaccination programs that involve fathers (rather than only mothers) in decisions about their children’s use of preventive healthcare services may lead to better levels of timely vaccine coverage.^112^ Similar to this, comprehensive analyses of research from low- and middle-income countries have shown the benefits of incorporating male partners in decisions regarding sexual and reproductive health, including family planning.^113^ As evidence from randomized controlled trials and other types of studies accumulates, a third strategy supporting interventions for gender transformation is becoming more and more credible.^114^ These aim to reshape male gender roles to support more equitable relationships between men and women. Such actions may strengthen sexually protective tendencies, stop intimate partner violence, alter gender-based views that are unfair, and lower the risk of STIs. Traditional health promotion interventions have failed to engage men. Men’s health promotion programs can be given in a variety of methods, but during the past several decades, the e-health sector has grown significantly ^115^. By granting end-user anonymity and liberty for selecting certain information, men’s e-health promotion programs can provide important chances to complement and link existing services as well as promote men’s behavioral changes and health promotion activities.^116^

 The exploration of men’s health programs reveals a multifaceted landscape where traditional interventions often fall short in engaging male participants. Recent studies highlight the effectiveness of alternative settings, such as sports clubs and workplaces, in promoting men’s health. ^117-123^

###  Sports Clubs as Health Promotion Settings

 Sports clubs offer male-friendly health promotion settings for men, but little is known about implementable intervention strategies and subsequent effects. Sports clubs have emerged as effective venues for health interventions, particularly for men with high cardiometabolic risks. These settings foster a sense of social identity, enhancing engagement and health behavior change. In a rapid realist review that identifies 52 studies on the context, mechanisms, and effects of health promotion interventions targeting men through sports clubs, a systematic literature search was conducted, focusing on studies published after 2013. Results showed that sports clubs can engage men with high cardiometabolic risk and produce favorable health outcomes. Strong attachment to the sports club resonates with men’s feelings of social identity, increasing their likelihood of program engagement and health behavior change. The study concludes that men’s health promotion through sports clubs is emergent, focusing on select behaviors and sub-groups.^124^

###  Workplace Health Promotion

 Workplace programs targeting smoking, nutrition, and physical activity show potential but yield mixed results. A systematic review found modest improvements in health behaviors, emphasizing the need for long-term studies to assess efficacy. This study evaluates workplace health programs targeting smoking, nutrition, alcohol, physical activity, and overweight and obesity risk factors in men. Results show encouraging results in interventions targeting overweight and obesity, physical activity, sugar-sweetened beverages, and snack consumption. However, fruit and vegetable consumption, smoking cessation, and alcohol consumption did not show significant differences. Personalized dietary feedback and technology could streamline weight loss interventions. In addition, this highlighted a slight decrease in body weight and BMI among participants, suggesting that workplace settings can benefit health promotion.^125^

###  Participatory Engagement

 Engaging men in the design and delivery of health programs is crucial. Health promotion interventions often struggle to engage men, with only 1/3 of participants being men. Gender-sensitized programs aim to tailor interventions to men’s needs, but increasing participatory engagement aligns with knowledge translation and implementation science recommendations. A systematic review found that while many interventions were tailored to men, only a third involved men in meaningful ways, indicating a gap in participatory engagement. Effective participatory methods can enhance health interventions’ implementation and sustainability, aligning with knowledge translation science recommendations.^126^ In contrast, while these findings underscore the potential of targeted interventions, they also reveal a persistent gap in addressing the diverse needs of men across different demographics and settings, particularly in underrepresented regions like sub-Saharan Africa.^127^

 Our systematic review of guidelines for men’s health programs revealed a strong emphasis on routine health screenings as a vital component of preventative care. This aligns with the growing recognition of the importance of early detection and intervention in improving men’s health outcomes. Prostate cancer was the most frequently discussed health issue, with 16 out of 23 guidelines recommending screening tests such as PSA and colonoscopy. This underscores the critical role of early detection in improving outcomes for men with prostate cancer. Guidelines also emphasized the importance of screening for and managing chronic conditions, including LUTS, STDs, mental health disorders, and neurocognitive disorders. This highlights the need for comprehensive approaches to men’s health that address both acute and chronic conditions. Promoting healthy lifestyles, including physical activity, smoking and alcohol cessation, and nutritional interventions, was a common theme in the guidelines. This emphasizes the importance of addressing behavioral risk factors in addition to medical interventions.

 The review provides a comprehensive overview of guidelines for men’s health, encompassing a wide range of health conditions and preventative measures. This offers a valuable resource for healthcare providers and policymakers seeking to implement evidence-based programs.^128^ The findings highlight the importance of routine health screenings as a vital component of men’s preventative care. This reinforces the need for early detection and intervention in improving health outcomes. It also underscores the importance of tailoring interventions to the specific needs and preferences of different groups of men. This emphasizes the need for a personalized approach to men’s health care. The emphasis on lifestyle interventions, such as physical activity, smoking and alcohol cessation, and healthy eating, aligns with current trends in preventative medicine. This highlights the importance of addressing both medical and behavioral risk factors.

 The emphasis on routine screenings can inform public health campaigns and policy initiatives aimed at improving early detection and prevention of diseases.

 The findings highlight the need for tailored interventions to address the specific needs of different groups of men. This can inform the development of culturally appropriate and effective programs.

 The emphasis on lifestyle interventions reinforces the importance of addressing behavioral risk factors in addition to medical interventions. This can inform public health campaigns and policies aimed at promoting healthy lifestyles.

 Gender equality initiatives should consider men’s health as a determinant, integrate gender budgeting, improve monitoring frameworks, and collaborate with social and educational sectors. Occupational safety regulations should be in place to address men’s exposure to high-risk occupations. Collaboration with civil society organizations and promoting transformative action in various contexts can improve men’s health. Health equity policies should also address social exclusion, particularly for men experiencing social exclusion due to age, ethnicity, sexual orientation, gender identity, homelessness, disability, or mental health difficulties. The identified guidelines and health conditions contribute to existing knowledge by providing specific recommendations for men’s health screenings and interventions. These findings are important for advancing public health practices and research as they highlight the need for tailored and evidence-based approaches to address men’s unique health needs.

 The research on men’s sexual health highlights several key areas of focus and intervention strategies aimed at improving health outcomes:

1. Motivational Interviewing: Effectively increases HIV counseling and testing rates among young African-American MSM.
2. Community Sensitization: Proven to prevent STDs by raising awareness and promoting safer sexual practices.
3. Family Planning Conversations: Engagement with health professionals about family planning significantly influences the use of modern contraceptives among sexually active men, as well as their reporting on partner contraceptive use.
4. Adult Circumcision and Risk Compensation: Men who underwent adult circumcision exhibited a 20% higher incidence of risky sexual behavior, indicating significant risk compensation.
5. Intervention Projects: A three-year initiative in Uganda positioned men as active participants in sexual and reproductive health, promoting them as clients, partners, and change agents.
6. Mobile-Based Educational Interventions: In Iran, such interventions for men with spinal cord injuries led to notable improvements in sexual adjustment and marital satisfaction.
7. Osteoporosis Education: Programs aimed at enhancing knowledge about osteoporosis showed potential benefits for both men and women in understanding prevention practices.

**Clinical Guidelines and Recommendations**

 Guidelines for male health emphasize routine health screenings for various conditions, including:

- Malignancies
- Lower urinary tract symptoms (LUTS)
- STDs
- Mental health issues
- Cardiovascular diseases
- Diabetes
- Osteoporosis
- Testosterone deficiency
- Hypertension
- Abdominal aortic aneurysm

Lifestyle factors are also crucial, with recommendations for:

- Regular physical activity
- Smoking cessation
- Alcohol abuse prevention
- Nutritional interventions

###  Rationale for Excluding Men Over 65 and Implications for Generalizability

 We restricted our review to men aged 20–65, focusing on those who face distinct barriers to help-seeking, often rooted in masculinity norms, workplace constraints, and limited interaction with PHC. This age group also shows the lowest utilization of preventive health services across global settings.

 However, we recognize that excluding older men does limit generalizability, as individuals over 65 face significant burdens from CVD, prostate cancer, neurocognitive disorders, metabolic disease, and multimorbidity. Many guideline recommendations, especially for cancer, osteoporosis, CVD, and cognitive screening, are most relevant to older adults. Therefore, the findings here primarily reflect HEP strategies for working-age men and may not apply to older age groups whose engagement barriers differ.

 Future evidence mapping should incorporate older men to better capture the full spectrum of educational needs and healthcare engagement challenges.

###  Influence of Methodological Quality on Interpretation

 Methodological quality across empirical studies was generally moderate, with limitations including small sample sizes, lack of long-term follow-up, and insufficient control for confounding in observational studies. Higher-quality studies, particularly RCTs tended to focus on decision aids for prostate cancer and motivational interviewing for HIV testing. The strongest evidence, therefore exists in a relatively narrow subset of men’s health concerns.

 Guidelines also showed moderate methodological quality using AGREE II, with variability in stakeholder involvement and rigor of development. Higher-quality guidelines more frequently addressed comprehensive preventive care, including CVD, diabetes, and mental health—areas less commonly evaluated in programs or empirical studies.

 Thus, the strength of the overall evidence base is uneven. Conclusions related to prostate cancer and HIV/STI education are supported by strong empirical studies, whereas conclusions regarding chronic disease education rely more heavily on guidelines and descriptive program reports.

 While this review provides valuable insights into the current state of guidelines for men’s health programs, it is important to acknowledge certain limitations. First, the quality of the included guidelines varied, which may affect the generalizability of the findings. Second, we included studies only on men 20-65 years old. We chose this demographic for several reasons. Health education programs for men aged 20-65 are crucial for addressing the unique health challenges they face, as well as for promoting overall well-being. This demographic often encounters various health risks, including chronic diseases, mental health issues, and lifestyle-related conditions. The implementation of targeted health education initiatives can significantly enhance men’s health outcomes and encourage proactive health management.

 The findings of this review can inform health education programs and policy development by prioritizing health screenings with emphasizing the importance of regular screenings for cancers, STDs, and mental health conditions; Tailoring interventions by developing programs that address the specific needs and concerns of different groups of men, such as those from marginalized populations or with particular risk factors; Promoting healthy lifestyles by incorporating education on physical activity, nutrition, and smoking and alcohol cessation into men’s health programs; Addressing gender-specific barriers by developing strategies to overcome barriers that prevent men from seeking healthcare, such as cultural norms and attitudes about masculinity.

 Future research should aim to address these limitations by conducting a more comprehensive review of guidelines from a wider range of countries and assessing the quality of the included studies. Additionally, studies evaluating the effectiveness of men’s health programs based on these guidelines would be valuable in informing future policy and practice.

###  Implications for Health Education and Policy

 Taken together, the findings demonstrate that successful HEPs for men share several characteristics:

- Delivery in male-friendly or community-relevant settings
- Peer-led or culturally tailored approaches
- Use of digital tools that support autonomy and privacy
- Emphasis on informed decision-making and practical action steps

At the same time, there is a clear need to diversify educational content beyond prostate cancer to address high-burden conditions such as CVD, diabetes, depression, and substance use—particularly for marginalized groups who face the highest risk of poor health outcomes.

###  Future Directions

####  Future studies should evaluate

- HEPs addressing underrepresented conditions (e.g., CVD, metabolic disease, mental health)
- Long-term behavioral and clinical outcomes
- Participant engagement across diverse demographic groups, including men > 65
- Intersectional approaches that integrate masculinity frameworks, cultural context, and structural determinants of health.

## Conclusion

 Based on a synthesis of health education programs, studies, and guidelines, this review concludes that effective strategies to engage men with health services are gender-sensitive, culturally tailored, and delivered in accessible, male-friendly settings (e.g., sports clubs, barbershops) using peer-led or digital approaches. A key finding is the disproportionate focus on prostate cancer screening in existing programs, which overlook other high-priority areas like cardiovascular disease, mental health, and lifestyle modification emphasized in clinical guidelines. To improve men’s health outcomes and equity, future efforts must diversify educational content to address this broader range of conditions, involve marginalized groups, and evaluate long-term impacts on health behavior and service use.

## Competing Interests

 None.

## Data Availability Statement

 Not applicable.

## Ethical Approval

 This study is part of a larger research project, which was approved by the Ethics Committee of Shahid Beheshti University of Medical Sciences, Tehran, Iran (IR.SBMU.RETECH.REC.1402.124). The review of text, which was a prerequisite for the overall study, was approved by the ethics committee of Tabriz University of Medical Sciences, Tabriz, Iran (IR.TBZMED.REC.1403.502).

## Supplementary Files

Supplementary File 1. Search strategy for Guidelines, programs, pamphlets, and original research.

Supplementary File 2. Characteristics of the original included studies in the systematic review
