# Supplementary material for: A Scoping Review of Health Education Programs for Improving Men’s Engagement with Health Services
Source: Health Promot Perspect. 2026 Aug 21;16(2):134–52. doi: 10.34172/hpp.45321 (PMC13723383; doi:10.34172/hpp.45321)
Supplement: Supplementary file 1 [file hpp-16-134-s001.pdf]

Hosseini et al, Health Promotion Perspectives 2026;16(2):134-S1
doi:10.34172/hpp.45321
https://hpp.tbzmed.ac.ir

Supplementary File 1. characteristics of the original included studies in the systematic review

Author, date	Origin	Aim	Study design	Population			Measures	Intervention/method of data gathering	Results
				Condition	Number	Age			
Zarei, 2020 [58]	Iran	To assess the effectiveness of a mobile-based educational intervention on sexo-marital life in Iranian men with spinal cord injury.	RCT	SCI	70	Experiment: 36.7; Control: 35.3	sexo-marital life; assessed through sexual adjustment, sexual satisfaction, marital adjustment, and marital satisfaction	A mobile application	The application-based educational intervention showed the positive effect of education on sexo-marital life in men with spinal cord injury.
Canuto, 2018 [54]	Australia	To identify the perceived motivators, barriers and enablers of Aboriginal and Torres Strait Islander men's utilization of primary health care services, explore their experiences and obtain suggestions from them as to	Ethnographic study	Aboriginal and Torres Strait Islander men	19	Not reported	Patients' experiences of health service use, including as a child	Semi-structured interviews	Feelings of invincibility, shame, being uncomfortable, fearful, along with long waiting times, having a lack of knowledge, and culturally inappropriate staff/services were all found to be barriers to service utilization.<br>Enabling factors included convenience, the perceived quality of the service, feeling culturally safe and/or a sense of belonging, and having a rapport with staff. Motivation for attending primary health care services included going when feeling sick/unwell, attending a particular service (dental or sexual health), visiting

		how services could be modified to improve utilization.							for check-ups and preventative health and family encouragement
Khani Jeihooni, 2021 [46]	Iran	To survey the effect of educational intervention based on health belief model and social support on testicular self-examination in men aged between 15 to 35 years of Fasa City, Fars province, Iran	Quasi-experimental study	Men	200 (100 in the experimental group and 100 in the control group)	27.26	A questionnaire consisting of demographic information, knowledge, health belief model (HBM) construct, and social support was used to measure testicular self-examination before, 3 months after the intervention, and 6 months later	6 educational sessions (testicular cancer, its prevalence and types, its risk factors, symptoms, infected areas, diagnosis, side-effects and its severity, understanding about testicular self-examination and its importance, benefits, and barriers of self-examination and correct way of doing TSE were discussed, role of social support).	
Roche, 2018 [59]	Australia	To explore the primary healthcare (PHC) needs and health service use of homeless men attending this clinic in inner Sydney, including men's views of the impact of	Multimethod design: a cross-sectional survey and adminis	Homeless men	cross-sectional survey: n = 40, administrative data	in cross-sectional: 51,	Health problems (self-report), primary health clinic treatments, services and referrals, per month other services and referrals	questionnaire administered at interview	Health problems reflected multimorbidity, with mental health issues present in almost all respondents. The majority had attended the clinic more than 20 times in the past year and said the services, treatments and referrals helped them avoid the emergency department. Administrative data indicated that medication administration was the most frequent service provided. Referrals to

		the care provided and approaches that may facilitate more effective care.	trative data		(n = 2707 daily summaries)				other health services doubled over the 7-year period.
Rizio, 2016 [60]	Australia	To pilot the delivery phase of an education program and evaluate a train-the-trainer approach for delivering men's health education to PHC nurses.		PHC nurses	facilitators =18, and peer workshop participants= 98	Not reported	The level of confidence in men's health and knowledge and skills in men's health promotion	8-h train-the-trainer workshop	After completing the train-the-trainer workshop, most facilitators expressed confidence (92%), and all indicated sufficient knowledge and access to resources to deliver a peer workshop. All agreed that the module was sufficiently flexible to suit their local setting. Following the peer education workshop, facilitators and workshop participants reported high levels of confidence and knowledge in men's health promotion
Holt, 2009 [48]	USA	To implement and provide an initial evaluation of a spiritually based prostate cancer screening informed decision making intervention for African-American (AA) men who attend church, and determine its efficacy for	Pre-post RCT	AA men	49	Not reported	Prostate cancer screening history, Knowledge, Prostate cancer beliefs, Self-efficacy, Perceived barriers to prostate cancer screening, Preparation for decision making, Acceptability/appropriateness	Spiritually based or the non-spiritual educational session	The spiritually based intervention appeared to be more effective in areas such as knowledge, and men read more of their materials in the spiritually based group than in the non-spiritual group

		increasing informed decision making.							
Mohammadian, 2020 [56]	Iran	To assess the effect of the educational intervention based of the theory of planned behavior (TPB) on manual material handling (MMH) Behavior	Quasi-experimental, pretest-posttest study	male soldiers	140	25.44	MMH attitude, Subjective norms in MMH, Perceived behavioral control in MMH, Behavioral intention in MMH, Behavior in MMH	Educational program based on the results for the amelioration of behavior regarding correct MMH	Educational intervention based on the TPB was effective in improving behavior for correct MMH in soldiers.
Ganassin, 2016 [57]	Brazil	To compare the knowledge about risk factors for cardiovascular diseases before and after an educational intervention involving male metal workers	Before-and-after RCT	male metal workers	135: Intervention group = 67, Control group = 68	40.3 , Intervention: 41.5, control: 39.1	Knowledge on risk factors for cardiovascular diseases based on the HDFQ - Heart Disease Fact QuestionnairK	A health education program	The educative intervention in group, at the workplace and during lunchtime, showed to be feasible and effective strategy to increase men's knowledge on risk factors for cardiovascular dis eases.
Capanna, 2015 [74]	Jamaica	To increase participants' knowledge about prostate cancer and increase the intention of men in the four parishes of	Pretest/ posttest study	men	454		Attitudes of participants towards healthcare providers and whether healthcare providers	Statistically significant improvements in the percentage of correct responses between the pretest and posttest were evident (p b 0.05). Additionally, screening rates in creased dramatically by 6 months post-intervention with over 33% of men receiving a prostate exam after partic ipating in the educational intervention. The theory-based educational intervention increased participants' knowledge of prostate cancer, types of screening tests, frequency	

		Western Jamaica to utilize screening services. Additionally, to determine the percentage that accessed prostate cancer screening by 6 months post-intervention during follow-up.					inform patients of prostate cancer screening, Participants' prostate cancer knowledge in pretest and posttest, Prostate cancer attitudes, beliefs and practices of men participating in the educational intervention	of screenings and risk factors and symptoms, and was effective in increasing screening rates among the men in Western Jamaica within 6 months post-inter ventionAn educational intervention	
Badger, 2013 [66]	USA	To examine selected survivor characteristics to determine what factors might moderate the response to two psychosocial interventions	RCT	prostate cancer survivors (PCSs)	71	66.99	Psychological quality of life (QOL) outcomes included depression, negative and positive affect, and perceived stress	telephone-delivered health education (THE) intervention or a telephone-delivered interpersonal counselling (TIP-C) intervention	For three of the psychological outcomes (depression, negative affect and stress), there were distinct advantages from participating in THE. For example, more favourable depression outcomes occurred when men were older, had lower prostate specific functioning, were in active chemotherapy, had lower social support from friends and lower cancer knowledge. Participating in the TIP-C provided a more favourable outcome for positive affect when men had higher education, prostate specific functioning, social support from friends and cancer knowledge.

Le, 2015 [49]	USA	To describe the development of the SMS text messaging component of the Men's Prostate Awareness Church Training (M-PACT) intervention. The overall M-PACT parent study aims to increase informed decision making for prostate cancer screening among African American men.	Cross sectional	AA men	288	56	Participant text message recall and acceptability ratings	SMS text messaging	At workshop 4, over 65percent of participants wished to continue receiving the messages. While there was an increase in recall over time, more than one-third of the participants did not recall receiving the 53 text messages. However, recall was considerably greater among men who attended the Men's Prostate Awareness Church Training workshops. Overall, the inclusion of text messages in health promotion interventions targeting mature African American men was found to be feasible and acceptable.
Barbosa, 2018 [61]	Brazil	To analyze the general aspects of adult men's access to PHC services	cross-sectional	Men	485	Not reported	General aspects of men's accessibility to health services, possibility of interference in the accessibility of men to health services, Issues related to male accessibility to PHC.	interviews	Men in the productive age group do not seek health services because they are unaware of the importance or lack of concern about health promotion and prevention actions, fear of illness, and institutional factors related to the organization of service hours of Family Health.

Vincent, 2018 [62]	Australia	To assess the likelihood of Australian men attending a dedicated men's health service (DMHS) and to better understand the reasons for their preferences and determine how health behaviours influence likelihood		men	1493	56	health service use and preferences, health help-seeking behaviours, and the likelihood of attending a DMHS	interviews	Seventy percent of men reported a moderate or higher likelihood of attending a DMHS. As young healthy men are more likely than older men to display health behaviours that are associated with a higher likelihood of attending a DHMS, such as delay/avoidance, marketing a DMHS to such men may be of value.
Okoro, 2020 [63]	USA	To (a) increase knowledge and risk awareness of targeted conditions, (b) change health-care-seeking attitudes toward regular primary care among AA men, and (c) improve their lifestyle-related health behaviors by leveraging the influence of	A pre-/post-intervention study	AA men	Men (n = 29), Women (n = 46)	Men: 45.1, Women: 49.5	Knowledge, Program Impact (Increased Knowledge/Awareness of Risk, Change in Health-Care-Seeking Attitudes, Increased Self-Efficacy to Engage the Health-Care System, Sense of Community and Social Support, Unique and	Educational Sessions	Major themes were—increased knowledge/awareness of risk associated with chronic conditions, change in health-care-seeking attitudes, increased self-efficacy to engage the health-care system, and lifestyle changes. Other impacts reported were building community/social support, a safe and enabling learning environment, and enhanced community health status overall

		women in their lives.					Enabling Learning Environment,		
Pringle, 2011 [107]	UK	To investigate the pre-adoption demographic and health profiles of men participating in a program of men's health delivered in English Premier League (EPL) football clubs		men	16	875	Self-reported lifestyle and health profiles including physical activity, consumption of fruit and vegetables, smoking, height, weight, consumption of alcohol and perception of health.	awareness-raising activity days for supporters, weekly healthy lifestyle classes, and outreach activities targeted in local communities	A national program of men's health promotion interventions delivered in EPL football clubs was effective in reaching target audiences. Interventions were predominantly adopted by men not meeting health guidelines
Pringle, 2013 [108]	UK	To investigate the impact of a national program of men's health delivered in/ by English Premier League (EPL) football clubs on health profiles.		men	1988	Not reported	health profiles including Activity (sessions/week), Sitting (hours/day), Daily portions of fruit/vegetables, Alcohol (units/week), BMI	educational activities on match days and weekly lifestyle classes at the football stadia/training venues	A national program of men's health delivered in EPL football clubs reached men failing to meet health guidelines. Interventions engaged men who neither consulted a GP nor used health information services.
Gross., 2016 [67]	Canada	To build solidarity and brotherhood among vulnerable men; to promote health through	mixed-methods design	DUDES Club members	150	46.8	Program satisfaction. Overall health cluster ratings. Benefits of	focus group interviews	Findings indicate that this innovative model is effective in promoting the well-being of mainly indigenous men through culturally safe services in an urban community

		education, dialogue, and health screening clinics; and to help men regain a sense of pride and fulfilment in their lives.					frequent attendance at DUDES Club meetings, Benefits of frequent attendance in each of the 4 medicine wheel clusters, Program benefits by ethnicity or cultural identification		
Chirau, 2012 [77]	Zimbabwe	To determine whether men who were circumcised in adulthood have risky sexual behaviour after being circumcised	Cross-sectional	men	131	29	The primary outcome indicators were incidence of sexual behaviors known to place men at increased risk of acquiring HIV. Secondary outcome indicators were incidence of STIs, satisfaction with the male circumcision procedure, attendance for review visits.	interviews	There was reduced prevalence of STI among the circumcised clients. Attendance for review visits was higher than national average. Clients were generally satisfied with the circumcision procedure, and there were contrasting opinions on its effect on sexual performance.

Ghanotakis, 2017 [109]	Uganda	To examine the extent to which the community based intervention component focused on male engagement achieved its aim of transforming harmful gender attitudes, norms, and behaviours among men participating in intervention activities	Cross sectional	male and female HIV C&T clients t	1122	42.1	The Gender Equitable Men (GEM) Scale	10 educational sessions	The Gender Equitable Men (GEM) Scale was used to assess the effect on gender attitudes. The intervention achieved negligible changes in responses to GEM items. Improvements in some gender-influenced health-seeking behaviours and practices in men were noted, specifically in visiting health facilities, HIV testing, and condom use.
Ganle, 2016 [64]	Ghana	To investigate and identify strategies for overcoming health system barriers to access and use of skilled delivery services in Ghana	Qualitative	pregnant women and lactating mothers and healthcare providers	?	?	Stakeholders proposed solutions to health system barriers	discussion or interview consisted of an open-ended thematic topic guide	Main themes emerging through interviews were (1) improving coverage and quality of services, (2) expanding free ambulatory services, (3) humanising hospitals and doctor–patient relationships, (4) promoting choice in skilled maternity care, (5) training and motivating more midwives and (6) building partnerships with traditional birth attendants (TBAs).

Stern, 2015 [33]	Uganda	To examined the impact of a three-year intervention project conducted in the Hoima district of Uganda, which sought to engage men in sexual and reproductive health as clients, equal partners and advocates of change	mixed-methods design	men, their female partners and male beneficiaries	164	?	Sexual and reproductive attitudes and practices of men, including those around STIs, HIV, contraception, abortion and gender equality.	quantitative surveys + Qualitative survey (the International Programmes Specialist from Sonke Gender Justice conducted the focus-group discussions and interviews)	Following the intervention, a significantly greater number of men accessed, and supported their partners in accessing sexual health services, had gained sexual and reproductive health awareness, reported sharing domestic duties and contraceptive decision-making, and displayed a decreased tolerance for domestic violence. It was more difficult to assess men's involvement and behaviours as advocates of change, which sheds light on the complexities of a gender transformative project and the importance of evaluating such projects from both men's and their partners' perspectives and at different levels of the male involvement model in sexual and reproductive health
Kabagenyi, 2014 [76]	Uganda	To examine the influence of discussing family planning with health workers on modern contraceptive use (MCU) among sexually active men, and their reports of partner contraceptive use	Secondary data analysis	sexually active men	1755	Not reported	Percent distribution of sexually active men by utilization of modern contraceptive methods in Uganda, Likelihood estimates of using modern methods of contraception	DHS men's questionnaire	The centrality of the role of discussion with health workers in predicting men's participation in family planning matters may necessitate creation of opportunities for their further engagement at health facilities as well as community levels. Men's discussion of family planning with health workers was significantly associated with modern contraceptive use.

		+ examine a series of individual level factors including the desire to have other children and the number of children surviving, to measure how these may ultimately influence men's contraceptive use.					among sexually active men in Uganda,		
Odeny, 2012 [110]	Kenya	To evaluate the effect of short message service (SMS) text messages on attendance of the scheduled seven-day postoperative visit after male circumcision for HIV prevention	RCT	men	1200			Text message	Text messaging resulted in a modest improvement in attendance at the 7-day post-operative clinic visit following adult male circumcision
Duncan , 2014 [68]	Australia	To examine the effectiveness of a 9-month IT-based intervention (ManUp) to improve the physical activity,	RCT	men	301	35-54 years; Print-based n=96 (mea(SE)43.84	Self-monitoring of physical activity and nutrition behaviors	ManUp, was based on social cognitive and self-regulation theories and specifically designed to target males.	A total of 301 participants completed baseline assessments, 205 in the IT-based arm and 96 in the print-based arm. A total of 124 participants completed all 3 assessments. There were no significant between-group differences in physical activity and dietary behaviors ($P \geq .05$).

		dietary behaviors, and health literacy in middle-aged males compared to a print-based intervention.				(0.59)); IT-based n=205 (mean(SE):44.17 (0.41))			Participants reported an increased number of minutes and sessions of physical activity at 3 months (exp(β)=1.45, 95% CI 1.09-1.95; exp(β)=1.61, 95% CI 1.17-2.22) and 9 months (exp(β)=1.55, 95% CI 1.14-2.10; exp(β)=1.51, 95% CI 1.15-2.00). Overall dietary behaviors improved at 3 months (exp(β)=1.07, 95% CI 1.03-1.11) and 9 months (exp(β)=1.10, 95% CI 1.05-1.13). The proportion of participants in both groups eating higher-fiber bread and low-fat milk increased at 3 months (exp(β)=2.25, 95% CI 1.29-3.92; exp(β)=1.65, 95% CI 1.07-2.55). Participants in the IT-based arm were less likely to report that 30 minutes of physical activity per day improves health (exp(β)=0.48, 95% CI 0.26-0.90) and more likely to report that vigorous intensity physical activity 3 times per week is essential (exp(β)=1.70, 95% CI 1.02-2.82). The average number of log-ins to the IT platform at 3 and 9 months was 6.99 (SE 0.86) and 9.22 (SE 1.47), respectively. The average number of self-monitoring entries at 3 and 9 months was 16.69 (SE 2.38) and 22.51 (SE 3.79), respectively. The ManUp intervention was effective in improving physical

									activity and dietary behaviors in middle-aged males with no significant differences between IT- and print-based delivery modes.
Friedman, 2009 [47]	USA	To explore the implications of applying Nutbeam's multidimensional health literacy framework to African American men's understanding of Prostate cancer (PrCA) information.		African American men	25	55.5 (±1.06) years (range 47–64 years)	functional health literacy was assessed using two modified Cloze tests and the Shortened Test of Functional Health Literacy in Adults (S-TOFHLA). Men also participated in interviews or focus groups during which they were asked questions about PrCA risk, prevention, and screening.	Phase One: Functional Health Literacy Assessment; Phase Two: Functional, Interactive, and Critical Health Literacy Assessment	Mean S-TOFHLA was 28.28 (±1.98), implying ''adequate'' comprehension. Mean Cloze was .71 (±.05) for a Grade 8 document and .66 (±.04) for a Grade 13 document, also showing ''adequate'' comprehension. Cloze scores for the Grade 8 resource were lower for participants with less education ($P = .047$). Despite having satisfactory literacy test scores, results from interviews and focus groups revealed participants' limited understanding and misconceptions about PrCA risk. Many wanted information about screening and family history delivered word-of-mouth by AA women and church pastors as few of them had ever received or actively sought out PrCA resources. Using Nutbeam's framework, gaps in health literacy which were not adequately captured by the validated tools emerged during the interviews and focus groups.
Victor, 2011 [53]	USA	To evaluate whether a continuous high blood pressure	Cluster RCT	black male	152	Intervention: 49.5 (2.4);	change in HTN control rate for each barbershop	standard BP pamphlets (8 shops, 77 hypertensive patrons per shop) or	The HTN control rate increased more in intervention barbershops than in comparison barbershops (absolute group difference, 8.8% [95% confidence

		(BP) monitoring and referral program conducted by barbers motivates male patrons with elevated BP to pursue physician follow-up, leading to improved HTN control				Control: 51.2 (2.6)	(Baseline and 10-month follow-up BP measurements and computer-assisted health interviews were conducted not by the barbers but rather by independently contracted, trained black field interviewers.)	an intervention group in which barbers continually offered BP checks with haircuts and promoted physician follow-up with sex-specific peer-based health messaging (9 shops, 75 hypertensive patrons per shop).	interval (CI), 0.8%-16.9%]) (P=.04); the intervention effect persisted after adjustment for covariates (P=.03). A marginal intervention effect was found for systolic BP change (absolute group difference, -2.5 mm Hg [95% CI, -5.3 to 0.3 mm Hg]) (P=.08).
Song, 2012 [69]	USA	To examine the relation between health-related quality of life (HRQOL) and health literacy among men with prostate cancer		Men with newly diagnosed clinically localized prostate cancer	1581	median 63 years	HRQOL was measured using the Short Form-12 General Health Survey (SF12; version 2.0). Health literacy was measured using the Rapid Estimate of Adult Literacy in Medicine (REALM).		Higher health literacy level was significantly associated with better mental well-being (SF12-Mental Component Summary [MCS]; P < .001) and physical well-being (SF12-Physical Component Summary [PCS]; P < .001). After controlling for sociodemographic (age, marital status, race, income, and education) and illness-related factors (types of cancer treatment, tumor aggressiveness, and comorbidities), health literacy remained significantly associated with SF12-MCS scores (P < .05) but not with SF12-PCS scores
Lepore, 2012 [52]	USA	To evaluate the efficacy of a decision support	RCT	Black men	490	55.04 years (SD	Demographics and medical history; Knowledge;	In addition to a condition-specific educational	Post-intervention, the intervention group had significantly greater knowledge, lower decision conflict, and greater

		intervention focused on prostate cancer testing in a sample of predominantly immigrant black men.				=6.29 years)	Decisional conflict; Verified physician visit to discuss testing; Testing intention, benefits-to-risk ratio of testing, and verified PSA testing; Congruence; Anxiety;	pamphlet, participants received a maximum of two tailored telephone education calls within one-month (median = 1 week) by trained graduate-level health educators (n=244), and the control group received fruit/vegetable education, n=246).	likelihood of talking with their physician about prostate cancer testing than the control group. There were no significant intervention effects on prostate specific antigen testing, congruence between testing intention and behavior, or anxiety. A tailored telephone decision support intervention can promote informed decision making about prostate cancer testing in black and predominantly immigrant men without increasing testing or anxiety.
Mohlala, 2011 [75]	South Africa	To assess the acceptability and feasibility of asking pregnant women to invite their Male sexual partners (MSPs) to attend antenatal clinic (ANC) and to take up VCT in the context of antiretroviral treatment (ART) provision and a community	RCT	pregnant women partner	1000	MSV PCT arm: 31 (0.32); MSP PIS arm: 30 (0.34)	sexual behaviour; Intimate partner violence;	pregnant women's acceptance of written invitations for VCT and pregnancy information sessions (PISs) (500 were randomly allocated to a partner's PIS invitation letter and the other 500 were allocated a partner's VCT invitation letter)	Thirty-five percent (175 of 500) pregnant women given voluntary counselling and testing (VCT) invitations for their partners brought their MSPs for antenatal clinic visit compared with 26% (129 of 500) given pregnancy information sessions (PISs) invitations [relative risk (RR) 1.36, 95% confidence interval (CI) 1.12–1.64, P = 0.002]. Thirty-two percent (161 of 500) MSPs in the VCT arm underwent HIV testing compared with 11% (57/500) in the PIS arm (RR 2.82, 95% CI 2.14– 3.72, $P < 0.001$). The proportions of women and men reporting unprotected sex during the pregnancy

		sensitization programme.							were lower in the MSP VCT arm than in the MSP PIS arm – 25 versus 81% (RR 0.30, 95% CI 0.22–0.42, P < 0.001) and 26 versus 76% (RR 0.34, 95% CI 0.25–0.47, P < 0.001), respectively. No differences were seen in intimate partner violence.
Myers, 1999 [45]	USA	To identify factors that predict adherence by African American men to prostate cancer education and early detection.	Clinical trial	African - American Men who did not have a personal history of prostate cancer.	548	40 –70 years	Early detection within 1 year of completing the baseline telephone survey	Minimal (n=221; mailed a letter and a reminder that invited them to a urology clinic for prostate cancer education and early detection) or an enhanced intervention group (n=192; were sent the same correspondence and were also given print material and telephone contacts, which were tailored to each recipient.)	Adherence was significantly higher (OR 5 2.6, CI: 1.7–3.9) in the enhanced intervention group than in the minimal intervention group (51% and 29%, respectively). Men who were age 50 years or older (OR 5 1.7, CI: 1.1–2.8), were married (OR 5 1.8, CI: 1.2–2.9), believed that prostate cancer early detection examination should be performed in the absence of symptoms (OR 5 2.3, CI: 1.3–4.0), and self-reported an intention to have an early detection examination (OR 5 1.9, CI: 1.2–2.9) were also more likely to adhere a package of print materials and telephone contacts tailored to each recipient can have a strong effect on the adherence behavior of African American men.
Myers, 2005 [50]	USA	To test the impact of an informed decision-making intervention on	RCT	African - American men	242	40-69 years	1) Complete screening (i.e., having a digital rectal exam (DRE)	Standard Intervention (SI) group (N=121) or an Enhanced	Overall, screening use was low among study participants. El group men had a screening frequency two times greater than that of SI group men, but the

		prostate cancer screening use.					and prostate specific antigen (PSA) testing), and 2) Complete or partial screening (i.e., having a PSA test with or without DRE).	Intervention (El) group (N=121). An informational booklet was mailed to both groups. El group men were also offered a screening decision education session.	difference was not statistically significant: 8% vs. 4% (OR=1.94) for complete screening, and 19% vs. 10% (OR=2.08) for complete or partial screening. Multivariable analyses showed that being in the El group and primary care practice were significant predictors of complete or partial screening (OR=3.9 and OR=5.64, respectively).
Myers, 2011 [73]	USA	To assess the impact of a mediated decision support intervention on primary care patient prostate cancer screening knowledge, decisional conflict, informed decision making (IDM), and screening.	RCT	Male patients eligible for prostate cancer screening	313 (SI:157; EI:156)	SI:56±5.0; EI:57±5.0	Change in knowledge and decisional conflict; informed decision making (IDM) and screening (with a permission to audio-record physician–patient encounters.)	participants were randomized to either an enhanced intervention (EI) or a standard intervention (SI) group. mailed brochure on prostate cancer screening	
Outlaw, 2010 [51]	USA	To determine whether field outreach with motivational interviewing, as compared with traditional field outreach, leads to	RCT	Young African American MSM	192 (96 MSM: motivational interviewing	19.79 (2.2)	agreeing to traditional HIV counseling and testing (an oral swab of the cheek) and returning for test results	Field outreach combined with motivational interviewing Vs. traditional field outreach	More of the participants in the motivational interviewing condition than the control condition received HIV counseling and testing (49% versus 20%; P=.000) and returned for test results (98% versus 72%;P=0.001). The addition of motivational interviewing to field outreach is effective in encouraging high-

		increases in HIV counseling and testing and rates of return for test results among young African American men who have sex with men (MSM).			; 92: traditional field outreach session)				risk young African American MSM to learn their HIV status. Also, peer outreach workers can be effectively trained to reduce health disparities by providing evidence-based brief counseling approaches targeting high-risk minority populations.
Partin, 2004 [55]	USA	To assess the effect of video and pamphlet interventions on patient prostate cancer (CaP) screening knowledge, decision-making participation, preferences, and behaviors.	RCT	Male veterans age 50 and older who had no CaP	1152 (usual care:384; Pamphlet:384; Video:384)	68.4	Patient knowledge (as assessed from a previously validated 10-item index), preferences (assessed from a single yes/no question regarding whether the patient thought they would have a PSA test in the next year), Patient participation (by a single question about whether CaP screening was discussed at their last clinic visit)), and decision-making outcomes; PSA testing rates (assessed at 2 weeks and 1 year post-target appointment using current procedural terminology codes stored in VA outpatient records)		
Pignone, 2013 [72]	USA and Australia	To compare three methods of values clarification for decision making about PSA screening in average-risk men ages 50-70 from	RCT	men	911	59.8 years	difference among groups in most important attribute, based on a single question postVCM; differences in unlabelled test	Three values clarification methods (VCM): 1) a balance sheet, 2) rating and ranking task, and 3) a discrete choice experiment (DCE).	Over 40% reported a PSA test within 12 months. Those who received the rating and ranking task (n= 307) were more likely to report reducing the chance of death from prostate cancer as being most important (54.4%), compared with either the balance sheet (n= 302, 35.1%) or DCE (n= 302, 32.4%) groups. (p< 0.0001) Those receiving the balance sheet were

		the US and Australia.				choice and intent to screen		more likely (43.7%) to prefer the unlabelled PSA-like option (as opposed to the “no screening”-like option) compared with those who received rating and ranking (34.2%) or the DCE (20.2%). However, the proportion who intended to have PSA testing was high and did not differ between groups (balance sheet 77.1%; rating and ranking 76.8%; DCE 73.5%, p = 0.731).
Holland, 2005 [65]	USA	To evaluate the effectiveness of patient and/or physician communication interventions to increase men’s utilization of preventive healthcare services.	men between the ages of 40 and 60 who were enrolled in a large southeastern insurance company’s health maintenance organiz	6677 (Chart Stickers (n = 3,339); No Chart Stickers (n = 3,338))	40-60	Colorectal cancer screening; cholesterol screening; prostate cancer screening; preventive care	A group of members whose provider would be receiving chart stickers, and one whose provider would not receive chart stickers. These two groups were further divided by randomly assigning members to four other groups: No Other Mailing, Letter and Pamphlet, Letter and Pamphlet followed by a Postcard, and Postcard Only.	Results showed that personalized communications that included health education for men combined with a patient-specific reminder system for providers led to a significant improvement in the number of men who received preventive health care screenings. Results also showed that communicating with the man’s loved ones in the home combined with a patient-specific reminder system for providers was significantly associated with improvement in preventive healthcare screenings. Further research should continue to evaluate the best methods for engaging men in the healthcare system.

				ation (HMO) and point of service (POS) products.					
Schapira, 2000 [71]	USA	To test the effect of a prostate cancer screening decision-aid on patients' knowledge, beliefs, and use of prostate cancer screening tests.	RCT	men who were receiving primary care at a Department of Veterans Affairs Hospital	257	50 to 80 years	Prostate Cancer Screening Knowledge; Prostate Cancer Screening Beliefs; Prostate Cancer Screening Decisions	prostate cancer screening decision-aid consisting of an illustrated pamphlet as opposed to a comparison intervention	The illustrated pamphlet decision-aid was effective in improving knowledge of prostate cancer screening tests: 95% of the experimental group were aware of the possibility of false-negative test results compared with 85% of the comparison group (P <.01). Ninety-one percent of the experimental group were aware of the possibility of a false-positive screening test result compared with 65% of the comparison group (P <.01). However, there was no difference in the use of prostate cancer screening between the experimental (82%) and comparison (84%) groups, (P >.05).
Wilt, 2001 [70]	USA	To evaluate whether a mailed educational pamphlet affected men's	RCT	men at least 50 years of age and did not report a history	550	mean age, intervention: 72.8 vs. 70.4 in	Patients' responses to a survey mailed 1 week after their clinic appointments; prostate-specific antigen (PSA)	"Early Prostate Cancer" pamphlet: Intervention Group: PSA education brochure mailed 7–10 days before appointment (n =	Respondents were predominantly elderly white men (mean age, 71 years; 90% white) with chronic illnesses (48% described their health as "fair" or "poor"). Men who received the educational pamphlet were better informed than men in the usual care group, as measured by

		knowledge about early detection of prostate cancer.		of prostate cancer.		control group	testing determined from electronic medical records.	275); Control Group: Usual care (n = 275)	correct responses to the following three questions about prostate cancer screening: the natural history of prostate cancer (32% vs. 24%; P = 0.10), whether treatment lengthens lives of men with early prostate cancer (56% vs. 44%; P = 0.04), and accuracy of PSA testing (46% vs. 27%; P < 0.008). The overall proportion of correctly answered questions was greater in the intervention group (45% vs. 32%; P < 0.001). Testing for PSA in the year after the index clinic appointments did not differ significantly between the intervention group and the usual care group (31% vs. 37%; P > 0.2).
Tung 2006 [78]	Hong Kong	To evaluate the effects on knowledge, health beliefs and preventive behaviours of an osteoporosis educational program for men.	RCT	All men aged 18 years old or above and able to read and write Chinese who attended the health	128	40·81 (13·46)	45-minute lecture, followed by a 30-minute video demonstration of weight-bearing exercises and then 15–30 minutes of open discussion. Samples of calcium-containing foods were showed to participants to enhance their understanding of the preventive strategies. In addition, a pamphlet about osteoporosis prevention was distributed for self-review of the lecture content. The OEP was delivered in Cantonese by a Registered Nurse and was conducted in small classes of between 10 and 15 men, and was repeated five times to accommodate all members of the intervention group.		The findings demonstrated the effects on increasing the knowledge and health beliefs in osteoporosis and its preventive behaviours, but not on enhancing self-efficacy in performing osteoporosis preventive behaviours.

				centre during the study period				
