# Supplementary material for: A Scoping Review of Health Education Programs for Improving Men’s Engagement with Health Services
Source: Health Promot Perspect. 2026 Aug 21;16(2):134–52. doi: 10.34172/hpp.45321 (PMC13723383; doi:10.34172/hpp.45321)
Supplement: Supplementary file 2 [file hpp-16-134-s002.pdf]

Hosseini et al, Health Promotion Perspectives 2026;16(2):134-S2
doi:10.34172/hpp.45321
https://hpp.tbzmed.ac.ir

Supplementary file 2

Search strategy for Guidelines

Database: Ovid MEDLINE(R) ALL / PubMed(R) <1946 to Present>
Search Strategy:
1 exp Men's Health/
2 ((men* or man* or male) adj3 (health* or well-being* or wellbeing*)).ab,hw,kf,kw,ti,tw.
3 Sexual Behavior/ or exp Sexual Health/ or Sex Education/
4 exp HIV/
5 exp Prostatic Neoplasms/
6 3 or 4 or 5
7 Male/
8 6 and 7
9 1 or 2 or 8
10 exp Practice Guideline/ or exp Guideline/
11 9 and 10
12 limit 9 to (guideline or practice guideline)
13 11 or 12

Search strategy for program, pamphlet, and original research.

Database: Ovid MEDLINE(R) ALL / PubMed(R) <1946 to Present>
Search Strategy:
1 exp Men's Health/

2 ((men* or man* or male) adj3 (health* or well-being* or wellbeing*)).ab,hw,kf,kw,ti,tw.
3 (health* adj3 education).ab,hw,kf,kw,ti,tw.
4 exp Health Education/
5 (self* adj3 (management or care*)).ab,hw,kf,kw,ti,tw.

6 exp Self Care/ or exp Self-Management/
7 3 or 4 or 5 or 6
8 1 or 2
9 7 and 8

10 (Package* or Container* or program* or Pamphlet* or Brochure* or Booklet*).ab,hw,kf,kw,ti,tw.
11 9 and 10
